# Hypophosphatasia

**DOI:** 10.1186/1750-1172-2-40

**Published:** 2007-10-04

**Authors:** Etienne Mornet

**Affiliations:** 1 Laboratoire SESEP, Centre Hospitalier de Versailles, Bâtiment EFS, 2 rue Jean-Louis Forain, 78150 Le Chesnay, France; 2 Equipe Structure et Fonction, EA2493, Université de Versailles Saint-Quentin en Yvelines, Versailles, France

## Abstract

Hypophosphatasia is a rare inherited disorder characterized by defective bone and teeth mineralization, and deficiency of serum and bone alkaline phosphatase activity. The prevalence of severe forms of the disease has been estimated at 1/100 000.

The symptoms are highly variable in their clinical expression, which ranges from stillbirth without mineralized bone to early loss of teeth without bone symptoms. Depending on the age at diagnosis, six clinical forms are currently recognized: perinatal (lethal), perinatal benign, infantile, childhood, adult and odontohypophosphatasia. In the lethal perinatal form, the patients show markedly impaired mineralization *in utero*. In the prenatal benign form these symptoms are spontaneously improved. Clinical symptoms of the infantile form are respiratory complications, premature craniosynostosis, widespread demineralization and rachitic changes in the metaphyses. The childhood form is characterized by skeletal deformities, short stature, and waddling gait, and the adult form by stress fractures, thigh pain, chondrocalcinosis and marked osteoarthropathy. Odontohypophosphatasia is characterized by premature exfoliation of fully rooted primary teeth and/or severe dental caries, often not associated with abnormalities of the skeletal system.

The disease is due to mutations in the liver/bone/kidney alkaline phosphatase gene (*ALPL*; OMIM# 171760) encoding the tissue-nonspecific alkaline phosphatase (TNAP). The diagnosis is based on laboratory assays and DNA sequencing of the *ALPL *gene. Serum alkaline phosphatase (AP) activity is markedly reduced in hypophosphatasia, while urinary phosphoethanolamine (PEA) is increased. By using sequencing, approximately 95% of mutations are detected in severe (perinatal and infantile) hypophosphatasia.

Genetic counseling of the disease is complicated by the variable inheritance pattern (autosomal dominant or autosomal recessive), the existence of the uncommon prenatal benign form, and by incomplete penetrance of the trait. Prenatal assessment of severe hypophosphatasia by mutation analysis of chorionic villus DNA is possible. There is no curative treatment for hypophosphatasia, but symptomatic treatments such as non-steroidal anti-inflammatory drugs or teriparatide have been shown to be of benefit. Enzyme replacement therapy will be certainly the most promising challenge of the next few years.

## Disease name and synonyms

• Hypophosphatasia

• Phosphoethanolaminuria

• Rathbun disease

• HOPS

## Definition and diagnostic criteria

Hypophosphatasia (OMIM 146300, 241500, 241510) is an inherited disorder characterized by defective bone and teeth mineralization and deficiency of serum and bone alkaline phosphatase (AP) activity.

## Epidemiology

The birth prevalence of severe hypophosphatasia was estimated to be 1/100 000 on the basis of pediatric hospital records in USA [1]. The incidence of moderate forms was never estimated but it is expected to be much higher, due to the number of patients with dominant forms carrying the same mutations than those found in recessive hypophosphatasia.

## Clinical description

Clinical expression ranges from stillbirth without mineralized bone to pathologic fractures developing only late in adulthood [2]. Depending on the age at diagnosis, six clinical forms are currently recognized: perinatal (lethal), infantile, childhood, adult, odontohypophosphatasia and a rare benign prenatal form characterized by *in utero *detection but much better prognosis than other prenatal forms (Table 1). However, it should be noticed that these clinical subtypes overlap, for instance infantile and childhood hypophosphatasia share some clinical symptoms, and patients with adult hypophosphatasia often had some clinical symptoms already in childhood.

**Table 1:** The six clinical forms of hypophosphatasia.

**Clinical form**	**Inheritance**	**Bone symptoms**	**Dental symptoms**	**Clinical diagnosis**
Perinatal lethal	AR	Hypomineralization Osteochondral spurs	na	Radiographs Ultrasonography
Prenatal benign	AD	Bowing of long bones Benign post-natal	na	Ultrasonography Clinical examination
Infantile	AR	Craniosynostosis Hypomineralization Rachitic ribs Hypercalciuria	Premature loss of deciduous teeth	Clinical examination Biology (serum AP activity, PEA and PLP). Radiographs
Childhood	AR (frequent) or AD (rare)	Short stature Skeletal deformity Waddling gait Bone pain/fractures	Premature loss of deciduous teeth	
Adult	AR or AD	Stress fractures: metatarsal, tibia Osteoarthritis	+/-	
Odontohypophosphatasia	AR or AD	Loss of alveolar bone	Exfoliation (incisors). Reduced thickness of the dentin. Enlarged pulp chambers of teeth. Dental caries	Clinical examination. Biology (serum AP activity, PEA and PLP).

na: not applicable;

AR : autosomal recessive;

AD: autosomal dominant.

In the ***lethal perinatal ***form, the patients show markedly *in utero *impaired mineralization. They have skin-covered osteochondral spurs protruding from the forearms or legs [3]. These spurs are often diagnostic for hypophosphatasia. Some infants survive a few days but have respiratory complications due to hypoplastic lungs and rachitic deformities of the chest. Other symptoms include apnea, seizures and marked shortening of the long bones. In the rare prenatal benign form, despite prenatal symptoms, there is a spontaneous improvement of skeletal defects.

In the ***prenatal benign ***form, despite prenatal symptoms, there is a spontaneous improvement of skeletal defects [4,5]. The patients manifest limb shortening and bowing and often dimples overlaying the long bones deformities, and some ultrasounds revealed progressive improvement of the skeletal deformities and mineralization during the third trimester of the pregnancy [4,6].

Patients with the ***infantile form ***may appear normal at birth; however, the clinical signs of hypophosphatasia appear during the first six months. This form also has respiratory complications due to rachitic deformities of the chest. Despite the presence of an open fontanelle, premature craniosynostosis is a common finding that may result in increased intracranial pressure. Radiographs show widespread demineralization and rachitic changes in the metaphyses. Hypercalcemia also is present, explaining in part a history of irritability, poor feeding, anorexia, vomiting, hypotonia, polydipsia, polyuria, dehydratation, and constipation. Increased excretion of calcium may lead to renal damage. In infants who survive, there is often spontaneous improvement in mineralization and remission of clinical problems, with the exception of craniosynostosis [7]. Short stature in adulthood and premature loss of deciduous teeth are also common, but the long-term outlook can be favorable [8].

Skeletal deformities, such as dolichocephalic skull and enlarged joints, a delay in walking, short stature, and waddling gait accompany the ***childhood form***. Signs of intracranial hypertension or failure to thrive are typical [2,9,10]. A history of fractures and bone pain usually exists as well. Focal bony defects near the ends of major long bones may be observed and help point to the diagnosis. Secondary metabolic inflammation seems to be common in the bone of patients [11] and hyperprostaglandinism affects the clinical severity [12]. Premature loss of dentition is common with the incisor teeth often being the first affected. Spontaneous remission of bone disease has been described, but the disease may re-appear in middle or late adulthood.

The ***adult form ***presents during middle age. The first complaint may be foot pain, which is due to stress fractures of the metatarsals. Thigh pain, due to pseudofractures of the femur, also may be a presenting symptom. There is also a predilection for chondrocalcinosis and marked osteoarthropathy later in life. Upon obtaining an in-depth history, many of these patients will reveal that they had premature loss of their deciduous teeth [13,14].

***Odontohypophosphatasia ***is characterized by premature exfoliation of fully rooted primary teeth and/or severe dental caries, often not associated with abnormalities of the skeletal system. The anterior deciduous teeth are more likely to be affected and the most frequent loss involves the incisors [15]. Dental X-rays show reduced alveolar bone, enlarged pulp chambers and root canals. Although the only clinical feature is dental disease, biochemical findings are generally indistinguishable from those in patients with mild forms of hypophosphatasia (adult and childhood). Odontohypophosphatasia should be considered in any patient with a history of early unexplained loss of teeth or abnormally loose teeth on dental examination [8].

## Etiology

The disease is due to mutations in the liver/bone/kidney alkaline phosphatase gene (*ALPL*; OMIM# 171760) encoding the tissue-nonspecific alkaline phosphatase (TNAP or TNSALP). TNAP is a phosphomonoesterase of 507 residues, anchored at its carboxyl terminus to the plasma membrane by a phosphatidylinositol-glycan moiety [16]. The enzyme is physiologically active in its dimeric form and cleaves extracellular substrates pyridoxal-5'-phosphate (PLP), phosphoethanolamine (PEA) and inorganic pyrophosphates (PPi). Its exact function in bone and dental mineralization is still unclear but involves hydrolysis of PPi [17], and perhaps mammalian-specific activities such as collagen [18] and calcium binding [19]. The *TNAP *gene is located on chromosome 1p36.1 [20] and consists of 12 exons distributed over 50 kb [21]. The gene is subject to high allelic heterogeneity [22] and more than 190 distinct mutations have been described [23]. Most of them (79%) are missense mutations. This variety of mutations results in highly variable clinical expressivity and in a great number of compound heterozygous genotypes.

### Genotype-Phenotype correlations

Attempts to assess the relative importance of missense mutations and the genotype-phenotype relationship were performed on the basis of clinical data from patients, transfection studies [24-35], computer-assisted modeling [19,27], and studies of the biochemical properties of AP in cultured fibroblasts of patients [36] or transfected cells [37]. These experiments allowed to study cell localization, degradation and alkaline phosphatase activity of mutant proteins. A good correlation was observed between the severity of the disease and *in vitro *enzymatic activity of the mutant protein [27,28,30,38]. Patients with mild hypophosphatasia carry at least one mutation that, when tested, exhibits significant residual enzymatic activity, while patients with severe hypophosphatasia carry mutations that, when tested, mostly do not product enzymatic activity. By using immunofluorescence and biochemical treatments, various mutations were characterized for their cell localization and their degradation [25,26,28,29,32-34,39,40]. These studies showed that most of the missense mutations found in severe hypophosphatasia produced a mutant protein that failed to reach the cell membrane, was accumulated in the *cis*-gogi and was subsequently degraded in the proteasome. By contrast, the missense mutations responsible for mild hypophosphatasia were found to be at least in part correctly localized to the cell membrane. By using the crystal structure of the *E. coli *alkaline phosphatase [41], and then the crystal structure of the human placental alkaline phosphatase [42], 3D models of the TNAP were built and used to localize the hypophosphatasia mutations in the molecule [19,27]. The severe missense mutations were shown to mostly affect residues localized in crucial domains of the protein while mutations found in mild forms affected residues more randomly dispatched on the molecule. Finally, and interestingly, the complementary approach consisting in *in vitro *alkaline phosphatase measurement, immunofluorescence, biochemical treatments and 3D modeling converged to give a view of the severity of a mutation (Table 2).

**Table 2:** 

**Class**	**Cell localization**	**Degradation, other features**	**3D model localization**	**Mutation and reference**	**AP activity (% wild type)**
1	Intracellular accumulation; fails to move beyond the *cis*-Golgi	Degradation in the proteasome	Functional domains (homodimer interface, calcium binding site, active site)	**R54C **(c.211C>T), [29]	0
				**N153D **(c.508A>G) [32]	0
				**I201T **(c.653T>C) [40]	0
				**E218G **(c.704A>G) [19]; [26]	0
				**D277A **(c.881A>C) [29]	0
				**D289V **(c.917A>T) [33]	0
				**G317D **(c.1001G>A) [25]	0
2	Intracellular accumulation; fails to move beyond the *cis*-Golgi	Degradation in the proteasome	Not localized in a particular domain	**A162T **(c.535G>A) [26]; [29]	18
				**G232V **(c.746G>T) [40]	34
3	Cell membrane and cytoplasme		Not localized in a particular domain	**F310L **c.979T>C [28]	72
				**E174K **(c.571G>A) [40]	88
				**I473F **(c.1468A>T) [40]	37
			Active site vicinity	**G438S **(c.1363G>A) [40]	71
4	Cell membrane		Not localized in a particular domain	**A115V **(c.395C>T) [34]	17
	Intracellular localization; absence on cell surface	Large-sized secretory protein lacking GPI; aggregation due to disulphide bonds beween new cystein residues		**c.1559delT **[35]	28
	Cell membrane	Disulphide bond between new cystein residues	Crown domain	**R433C **(c.1348C>T) [39]	4

Attempt to classify the ALPL gene mutations according to site-directed mutagenesis, in vitro alkaline phosphatase activity assays, and cell localization by immunofluorescence. Only mutations studied for all these parameters are shown. Class 1 represents the most severe mutations resulting in mutant proteins accumulated in the cytoplasm, subsequently degraded, and therefore producing no in vitro residual activity. These mutations affect residues of functional domains of the enzyme and were mostly found in patients with severe hypophosphatasia. Mutations of class 2 are also accumulated in the cell but exhibit low but significant in vitro residual activity and could be therefore degraded with delay. These mutations, that do not affect particular functional domains of the protein, must be also considered as severe alleles. Mutations of class 3 are in part accumulated in the cytoplasm but also in part reach the cell membrane. They exhibit high in vitro residual activity and except G438S, do not affect residues of functional domains. These mutations are observed in patients with mild forms of hypophosphatasia. Class 4 regroups particular mutations not assignable to the above classes.

Nucleotides numbering is given according to [77] and the Nomenclature Working Group [78]: the first nucleotide (+1) corresponds to the A of the ATG initiation codon. Amino acids numbering is given according to a non-standardized nomenclature [77] taking into account of a 17-residues signal peptide,*i.e. *the ATG initiation codon is numbered as residue minus (-)17.

### The dominant effect of TNAP mutations

Dominant transmission of hypophosphatasia has been suggested on the basis of pedigree and laboratory data [13,43-45]. More recently, mutations responsible for this condition were identified: c.1133A>T (D361V) [46,47], c.346G>A (A99T) [48-50], c.188G>T (G46V), c.550G>T (R167W) and c.1433A>T (N461I) [49], c.323C>T (P91L) and c.1240C>A (L397M) [50], c.1259G>C (G403A), c.1402G>A (A451T) and c.1427A>C (E459A) (our unpublished data). *In vitro*, these mutations were shown to inhibit the normal monomer in the heterodimer made of mutant and normal proteins, resulting in decreased levels of alkaline phosphatase activity. Instead of the 50% expected in heterozygotes, alkaline phosphatase activities were found to range from 20% to 40% of wild-type [49]. The most strong *in vitro *inhibition was found with mutations D361V and G46V, two mutations responsible for the benign prenatal form of hypophosphatasia. Interestingly, parents of patients affected with benign prenatal hypophosphatasia express only very mild symptoms (mostly premature loss of teeth) or even, may be completely unaffected [4,5,47]. This is also the case of families with mild hypophosphatasia due to dominant missense mutations. So, dominance is sometimes difficult to demonstrate by using familial analysis, since expression of the disease may be highly variable, with parents of even severely affected children showing no or extremely mild symptoms of the disease [2,4]. This may be attributable both to the progressive improvement of affected patients from infancy to adulthood [13,36,51,52] and to epigenetic factors involved in the variable expression of the disease. It is possible that in particular stages of development alkaline phosphatase requirements are beyond the capacity of the heterozygous cell, resulting in hypophosphatasia symptoms. Then, AP requirements may be less important and filled by the heterozygous cell, which may explain the improvement in adult patients. It is also possible that the maternal alkaline phosphatase plays a role *via *fetal-maternal exchanges, as suggested by the prenatal benign form that seems to be observed only when the mutation is inherited from the mother [4-6].

## Diagnostic methods

In addition to clinical and radiographic examinations (see clinical description), hypophosphatasia diagnosis is based on laboratory assays, and since 1990s, molecular biology which appears to be very effective.

### Laboratory assays

Total serum AP activity is markedly reduced in hypophosphatasia. Thus, the diagnosis can be suggested in individuals in whom serum AP activity is clearly and consistently subnormal. In general, the more severe the disease, the lower the serum AP activity level appropriate for age [2]. However, AP activity is only a helpful diagnostic indicator as other conditions may also show this finding: early pregnancy, drug administration, hypothyroidism, anemia, celiac disease *etc*. It must be also noticed that serum AP dramatically varies with age and sex.

Increased urinary phosphoethanolamine (PEA) levels supports a diagnosis of hypophosphatasia but is not pathognomonic. It is also observed in a variety of other conditions, including several metabolic bone diseases, and some hypophosphatasia patients may have normal PEA excretion. In fact, the demonstration that PEA is also a natural substrate of TNAP *in vivo *remains to be confirmed [53].

Increased pyridoxal 5'-phosphate (PLP) may be a sensitive marker for hypophosphatasia. [2].

Heterozygous carriers of the severe forms are usually clinically normal but often show modestly reduced serum AP activity and increased urinary PEA [54].

### Molecular biology

Screening for mutations in the *TNAP *gene is essential to confirm the hypophosphatasia diagnosis when biochemical and clinical data are not clear enough, to offer genetic counseling or to offer molecular prenatal diagnosis to families affected by severe forms of the disease (see below). Clinical and biochemical data may not always distinguish hypophosphatasia from other skeletal diseases such as osteogenesis imperfecta. Mutation screening may be performed by single-stranded conformation polymorphism (SSCP) or denaturing gradient gel electrophoresis (DGGE) followed by sequencing of exons exhibiting variants [55-62], by direct sequencing of the cDNA [36,46,63] or by direct sequencing of genomic sequences [30,64-67]. The exons are small and few in number, making relatively easy the analyze. However, the fact that the mutations are spread over all the exons often means that the whole coding sequence has to be analyzed. In addition, some mutations remain undetectable despite of exhaustive sequencing of the coding sequence, intron-exon borders and untranslated exons. This may be due to mutations lying in intronic or regulatory sequences, but also to the expression of heterozygous mutations, especially in moderate (childhood, adult and odonto-) hypophosphatasia. By using sequencing, approximately 95% of mutations are detected in severe (perinatal and infantile) hypophosphatasia, while patients with mild forms often carry only one detected mutated allele. This may be due to expression of the disease at the heterozygous state in some of these patients.

## Differential diagnosis

• Osteogenesis imperfecta

• Rickets

• Achondrogenesis

## Antenatal diagnosis

Prenatal assessment of severe hypophosphatasia may be performed in couples with a previous affected child or a previous affected pregnancy. Mutation analysis of chorionic villus DNAs is now well documented [68-71] and is routinely performed in few laboratories. It seems that mutation analysis is more reliable than AP assay of chorionic villus sampling at least for heterozygote detection where low AP values may be misinterpreted [71]. Prenatal and postnatal diagnoses were also reported by using linked or intragenic polymorphisms [20,72]. In pregnancies with clinical symptoms detected by ultrasound but no familial history of hypophosphatasia, the prenatal diagnosis by mutation analysis remains possible. However, such analyze is difficult, due to the time needed for the *ALPL *gene sequencing, and may not always lead to a result.

## Genetic counseling

Genetic counseling of hypophosphatasia is complicated by the inheritance that may be autosomal dominant or autosomal recessive, the existence of the uncommon prenatal benign form [4,5], the variable expression of the disease in heterozygotes, the probable effect of *ALPL *gene polymorphisms, and the possible effect of mutations and polymorphisms of other genes that may modulate the hypophosphatasia phenotype (modifier genes).

Severe forms of the disease (perinatal and infantile) are transmitted as an autosomal recessive trait, while both autosomal recessive and autosomal dominant transmission have been shown in clinically milder forms [13,43-45]. Therefore, the risk of recurrence of severe forms is 25%. In moderate forms, it may be 25% (recessive transmission), 50% (dominant transmission) or still different (less than 50%) due to the variable expressivity of dominant forms [49,50]. The mutations detected in dominant forms and responsible for moderate hypophosphatasia are also found in severe recessive hypophosphatasia, associated to other mutations [48-50]. These mutations have a dominant negative effect due to the inhibition of AP activity of the wild-type/mutant heterodimer [47,49], or due to intracytoplasmic sequestration of the heterodimer [Lia-baldini *et al.*, in preparation]. Testing patient's relatives is useful since heterozygotes may express a mild form of the disease. In regard to the frequency of the disease, testing spouses of carriers is not primordial unless there is an history of consanguinity.

## Management including treatment

There is no curative treatment of hypophosphatasia, but symptomatic treatments are starting to be used in addition to orthopedic management. Treatments with zinc and magnesium (catalytic ions of the enzyme), and pyridoxal 5'-phosphate were reported to not significantly improve the patient's condition. However, the high clinical heterogeneity and the fact that the disease is rare make almost impossible controlled clinical trials. Preliminary results suggest that dietary phosphate restriction could be helpful in hypophosphatasia [73]. Non-steroidal anti-inflammatory drugs were shown to significantly improve the clinical features of childhood hypophosphatasia, especially in regard to pain [12,74] and to the secondary metabolic inflammation resulting from the disease [11]. Teriparatide (the recombinant human parathyroide hormone PTH 1–34) was successfully used to improve and resolve metatarsal stress fractures in adult hypophosphatasia [62].

In 1997, MP Whyte's group (Saint-Louis, MI, USA) attempted to treat an 8-month-old girl affected with highly severe hypophosphatasia by bone marrow cell transplantation [75]. The patient was given T-cell-depleted, haplo-identical marrow from her healthy sister, and significant and prolonged clinical and radiographic improvement were observed. Another 9-month-old girl suffering from similar course of infantile hypophosphatasia was treated by using bone fragments and cultured osteoblasts [76]. Seven years after transplantation, the patient was reported to be active and growing, and having the clinical phenotype of the more mild childhood form of hypophosphatasia [76]. These results suggest that donor bone fragments and marrow may provide precursor cells to form TNAP replete osteoblasts that can improve mineralization [75,76]. Another interesting way of treatment would be to act onto the expression of the plasma cell membrane glycoprotein-1 (*PC-1*) gene, an antagonist of the *TNAP *gene [17]. Indeed, it has been shown in mice that inactivation of the *Pc-1 *gene in *TNAP*-knock-out mice allows to restore the normal bone phenotype [17]. Finally, enzyme replacement therapy by using a substitutive enzyme targeting mineralized tissue should be the most promising challenge of the next few years.

## Prognosis

The perinatal form is almost always lethal within days or weeks, and around one half of patients with the infantile form dye from respiratory complications. Longevity studies were not reported in the infantile and childhood forms. Patients affected with adult or odontohypophosphatasia are believed to have normal lifespan.
